# Speckle-Based Sensing of Microscopic Dynamics in Expanding Polymer Foams: Application of the Stacked Speckle History Technique

**DOI:** 10.3390/s21206701

**Published:** 2021-10-09

**Authors:** Dmitry Zimnyakov, Marina Alonova, Ekaterina Ushakova, Sergey Volchkov, Olga Ushakova, Daniil Klimov, Ilya Slavnetskov, Anna Kalacheva

**Affiliations:** 1 Physics Department, Yury Gagarin State Technical University of Saratov, 77 Polytechnicheskaya st., 410054 Saratov, Russia; alonova_marina@mail.ru (M.A.); katushakova96@yandex.ru (E.U.); volchkov93@bk.ru (S.V.); s_sov@rambler.ru (O.U.); daniil.klimov.1999@mail.ru (D.K.); slawnetzkov@yandex.ru (I.S.); kalachova.anna98@yandex.ru (A.K.); 2 Precision Mechanics and Control Institute of Russian Academy of Sciences, 24 Rabochaya st., 410024 Saratov, Russia

**Keywords:** dynamic speckles, multiple scattering, stacked speckle history, polylactide foam, boiling, translation

## Abstract

Microscopic structural rearrangements in expanding polylactide foams were probed using multiple dynamic scattering of laser radiation in the foam volume. Formation and subsequent expansion of polylactide foams was provided by a rapid or slow depressurization of the “plasticized polylactide–supercritical carbon dioxide” system. Dynamic speckles induced by a multiple scattering of laser radiation in the expanding foam were analyzed using the stacked speckle history technique, which is based on a joint mapping of spatial–temporal dynamics of evolving speckle patterns. A significant decrease in the depressurization rate in the case of transition from a rapid to slow foaming (from 0.03 MPa/s to 0.006 MPa/s) causes dramatic changes in the texture of the synthesized stacked speckle history maps. These changes are associated with transition from the boiling dynamics of time-varying speckles to their pronounced translational motions and are manifested as significant slopes of individual speckle traces on the recovered stacked speckle history maps. This feature is interpreted in terms of the actual absence of a new cell nucleation effect in the expanding foam upon slow depressurization on the dynamic scattering of laser radiation.

## 1. Introduction

Over the past four decades, speckle-based probing techniques have been established as effective tools for characterizing media with complex structures and dynamics at a microscopic level. Starting with the pioneering works [1,2,3], a huge number of experimental and theoretical works have been carried out on various aspects of speckle diagnostics of the random multiple scattering media. One of the fundamental principles of speckle-based probes of microscopic dynamics in the diagnosed media is based on the analysis of temporal decorrelation of the intensity of a speckle-modulated light field at a fixed detection point. This time-varying speckle field is formed as a result of multiple scattering of the probe laser radiation in the diagnosed medium. The correlation time of speckle intensity fluctuations depends on the characteristic time scale of the scatter displacement to a distance of the order of the probe light wavelength and average number of scattering events in the medium. Application of the “classical” single-point-detection version of speckle correlation analysis, used in the early works on diffusion-wave spectroscopy of the random dynamic media, is limited by certain requirements for detection conditions and properties of the diagnosed media. First, during the sampling of the speckle intensity data, the probed medium must allow the consideration as a stationary and ergodic system (or, at least, as a system with weak non-ergodicity and non-stationarity). Second, the size of the detection zone should be less than the characteristic speckle size in the detection plane; violation of this condition will lead to a decrease in the speckle modulation depth of the detected optical signal.

At a later stage in the development of speckle-based sensing techniques (from the mid-1990s to the mid-2000s), new effective approaches to the analysis of dynamic speckle patterns were developed. These approaches were based on the principle of simultaneous detection of speckle-modulated optical signals from numerous different statistically independent regions of the dynamic speckle pattern. Consequently, the use of a multi-element matrix or linear photodetectors is assumed. In this regard, it is worth mentioning such probing techniques as the multi-speckle diffusing-wave spectroscopy [4,5], the Laser Speckle Contrast Analysis (LASCA) proposed by J.D. Briers et al. for visualization and characterization of blood microcirculation in human tissues [6,7], etc. All of these methods assume equivalence of the statistical characteristics of the speckle pattern obtained by averaging the one-time intensity values over the speckle ensemble (spatial averaging) and averaging over time for one speckle (i.e., ergodicity or at least weak non-ergodicity of the pattern).

It should be noted that an approach based on the synthesis of spatial–temporal speckle textures can be applied to visualize and analyze non-stationary rapidly changing speckle fields. Within the framework of this approach, the captured sequence of speckle images (frames) is transformed to a single image by extracting a row or column with a given number from each frame and combining them in the order of increasing frame numbers. Such transformation can be defined as generation of a “stacked speckle history”. In the late 1980s, synthesis of the stacked speckle history (SSH) maps was applied to visualize a gradual drying of ex vivo plant specimens [8]; scattering of a laser beam in a dried specimen causes formation of a speckled light field characterized by a slowly decaying spatial–temporal dynamics. The SSH mapping was also applied by D. Duncan and S. Kirkpatrick in the speckle-elastographic probing of biological tissues [9]. The texture of the synthesized SSH maps contains information about peculiarities of spatial–temporal dynamics of the detected speckles (in particular, their spatial–temporal correlations).

Beginning with the classical works on speckle metrology of solid body displacements carried out in the eighties of the last century (see, e.g., [10,11,12]), it is customary to distinguish two extreme cases of spatial–temporal dynamics of non-stationary speckle fields in any observation plane, such as translational dynamics and boiling dynamics. Ideal conditions for observing translational dynamics of speckles are associated with in-plane speckle detection in the far diffraction zone when steady-structured surface- or volume-scattering objects are illuminated by a plane coherent wave during displacement in the direction perpendicular to the wave vector of the illuminating wave. In this case, the speckle pattern in the observation plane is displaced parallel to itself without any changes in the structure at the microscopic level (on a spatial scale of the order of the speckle size). The speckle displacement rate is rigidly related to the object displacement velocity via the detection geometry. The opposite extreme case corresponds to the so-called boiling dynamics of speckles and is observed when laser radiation is scattered in the systems with completely stochastic particle motion of scattering sites without any drift component (for example, in the ensembles of Brownian scatterers).

Among the variety of the multiple scattering media with a complex structure and dynamics that can be probed using speckle-based techniques, foam-like systems are of partial interest due to their wide spread in various fields of human activity. From the point of view of statistical optics, any foam is a specific case of the multiple scattering two-phase random media. The scattering sites in non-light-absorbing liquid or polymeric foams are associated with the following objects randomly distributed in the foam volume: the walls of gas bubbles, the zones of wall intersections (Plateau–Gibbs channels, PGC), and nodes of the random PGC grid. Within the framework of the concept of multiple scattering of coherent radiation by the random media, the key influence on the stochastic phase modulation of the propagating light (and, accordingly, on the formation of speckle structures in a scattered light field) is exerted by a limited set of parameters. These parameters are the relationship between the characteristic size of the scattering medium L and the transport mean free path (MTFP) of light propagation in the medium $l^{*}$, as well as the scattering anisotropy parameter g of the medium (see, e.g., [13]). The latter parameter characterizes an ensemble-averaged angular distribution of scattered radiation in a single scattering event and tends to zero for the ensembles of small-sized Rayleigh scatterers. On the contrary, for the media consisting of large scattering sites with the sizes larger than the wavelength, the scattering anisotropy parameter asymptotically tends to a unity with an increase in the scatterer size. For a given value of g, the MTFP value is determined by such structural characteristics of the medium as the characteristic size of the scattering sites and their volume fraction in the medium. From the general considerations, it can be concluded that for the foam-like media, the transport mean free path should in a certain way correlate with the average size of gas bubbles 〈D〉 in the foam volume. Systematic experimental studies of optical transport properties of metastable liquid foams, carried out in the beginning of the 2000s by D. Durian et al. [14], made it possible to establish a close to linear relationship between $l^{*}$ and 〈D〉 with the proportionality coefficient determined by the volume fraction of a liquid phase in the foam. Thus, rapidly expanding unstable foams should demonstrate strong changes in the optical transport parameters in the course of evolution, accompanied by a pronounced dynamics of speckle structures in laser radiation scattered by the foam.

Polymeric foams are of particular interest among other foam-like media due to their extensive use in various modern scientific and industrial applications (beginning from the construction industry and ending with biomedical applications). For instance, highly porous polymer matrices are actively used in tissue engineering and regenerative medicine as a material platform for scaffold fabrication [15,16,17]. The trend in the synthesis of functional materials for biomedical applications has emerged over the past three decades, and one of the possible directions of scaffold fabrication is foaming of raw biocompatible and bioresorbable polymers (for instance, polylactic acid) using supercritical fluid agents (typically, carbon dioxide) [18,19,20,21,22,23,24,25,26,27]. This technology includes the following main stages:

− Plasticization of the initial polymer for the required time interval in the atmosphere of subcritical or supercritical carbon dioxide at a given temperature and pressure;
− Foaming of the plasticized polymer as a result of the changes in the pressure (depressurization) and temperature in the reactor according to a given scenario;
− Stabilization of the structure of the synthesized porous matrix.

In addition to physicochemical properties of the raw polymer, the control parameters in this technology include the initial values of pressure and temperature during plasticization, as well as the rate of pressure release at the stage of depressurization [23,25,27,28,29,30]. Depending on these parameters, the structural properties of the synthesized highly porous matrices (the average size and shape of pores, their volume fraction in the matrix, fraction of open pores, degree of interconnectivity, etc.) occur in wide ranges. Until now, the relationship between the control parameters in the foaming technology and structural characteristics of the synthesized matrices was established purely empirically as a result of sequential choice of parameter values and a posteriori analysis of the structure of produced matrices using traditional methods (the X-ray computed tomography, the scanning electron microscopy, etc.). Probing of an intensive foam growth at the microscopic level in a high-pressure reactor during depressurization is a complex experimental problem that has not yet been resolved. In this case, the traditional methods mentioned above are inapplicable since it is impossible to provide remote access to the object in the reactor and there is a need for special preparation of probed samples. At the same time, such probing is crucial not only for a further development of the foaming technologies, but also for establishing fundamental features of the transient processes in such substantially nonequilibrium and nonstationary systems as evolving polymer foams. Speckle-based probing techniques are one of the few approaches to effectively resolve this problem, and the use of additional capabilities in the analysis of spatial–temporal dynamics of speckle-modulated scattered laser radiation makes it possible to expand the functionality of the probing procedure.

Accordingly, the aim of this work was to adapt the SSH mapping technique to sensing the structural rearrangements in expanding polymer foams during the synthesis of highly porous polymer matrices and, on this basis, to identify the fundamental features in the behavior of evolving foams depending on the foaming conditions.

## 2. Materials and Methods

### 2.1. Experimental Technique

The experiments were focused on the study of spatial–temporal dynamics of speckle-modulated laser radiation multiple scattered by evolving polylactide foams. The studied samples of the expanding foam were obtained using depressurization of the “amorphous D,L-polylactide–carbon dioxide” system in a multi-window high-pressure reactor. This technique, previously described in [28,29,30], was developed for the synthesis of highly porous polylactide matrices as prototypes of the scaffolds for the regenerative medicine and tissue engineering. Typically, the synthesis includes the following basic stages:

− Preliminary plasticization of the raw granular D,L-polylactide due to exposure in the atmosphere of subcritical or supercritical carbon dioxide at a given pressure and temperature;
− Depressurization of the obtained solution of carbon dioxide in the polymer according to a given scenario, accompanied by nucleation (formation of an ensemble of bubble nuclei in the plasticized polymer) followed by the transition to intensive expansion of the polymer foam;
− Stabilization of the structure of the synthesized highly porous matrix at low pressures when transition to a glassy state of the polylactide occurs.

The second stage is crucial from the point of view of formation of highly porous matrices with the required structural characteristics (the average pore size, their volume fraction in the matrix, and the expansion factor determined by the ratio of the matrix volume to the initial polymer volume). It should be noted that nucleation and subsequent expansion of the foam have a pronounced stochastic character and are controlled by a number of factors, some of which compete with each other. Moreover, contributions of various factors (such as viscosity of the polymer, the surface energy of the polymer/carbon dioxide interfaces, the diffusion coefficient of carbon dioxide in the polymer, etc.) are time dependent during depressurization. Therefore, development of a speckle-based sensor system that makes it possible to track the features of formation of the structure of the polymer foam is a rather urgent and timely problem.

**Figure 1:**
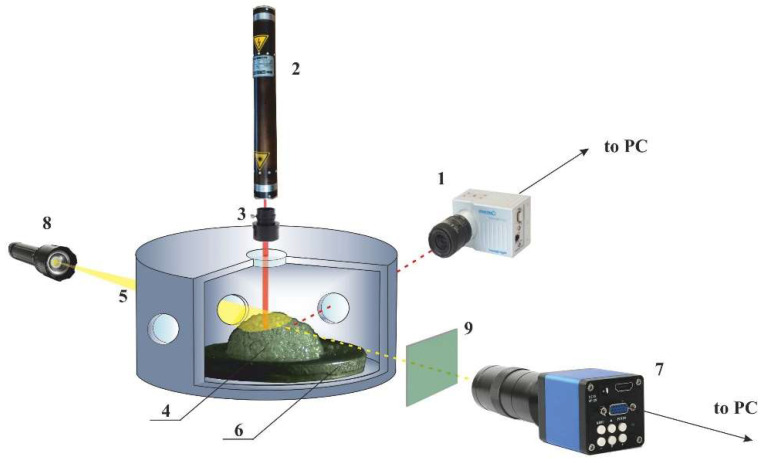
The scheme of the experimental setup.

Figure 1 is the schematic of the setup used in the experiments to study spatial–temporal dynamics of the speckle-modulated laser radiation scattered by expanding polylactide foam samples. Note that the elements of the high-pressure system (plasticizing/foaming agent container, high pressure pump, inlet and outlet capillaries, valves, etc), heaters, and pressure and temperature gauges are not shown in the diagram. The reader can refer to [30] for more details regarding the assembly of these units. A collimated laser beam with the wavelength of 633 nm was incident through the upper window of a multi-window high-pressure reactor onto the sample of expanding polylactide foam in the reactor. The laser radiation multiple scattered in the foam volume was captured through the side window of the reactor using a high-performance CMOS camera (1), Optronis CamRecord CR3000 × 2 type (Optronis GmbH, Kehl, Germany)). A He-Ne laser (Plasma company, Ryazan city, Russia) (2) with the output power of 2 mW and linear polarization of the beam was used as an illumination source; the diffraction divergence of the laser beam was reduced using a collimator (ThorLabs, Newton, NJ, USA) (3). The diameter of the beam falling onto the foam sample (4) was equal to 3 mm. A specially designed prototype of the high-pressure reactor (5) was made of stainless steel and was equipped with six symmetrically positioned side windows and one top window made of sapphire glass; the diameter of the windows was equal to 8 mm. The design of the reactor allowed us to provide the plasticization stage at the pressures up to 25 MPa.

Weighed portions of D,L-polylactide used for foaming were placed in a metal cylindrical container 6 made of stainless steel; the inner diameter of the container was 10 mm and the height of the container was 1 mm. Accordingly, the volume of polylactide specimens in the sample container before foaming was ≈80 mm^3^.

The required fixed temperature in the reactor in the range from 20 °C to 50 °C was provided using resistive heaters covering the side surface of the reactor (except for the windows) and a thermocouple-based feedback system. A K-type thermocouple located at the bottom of the reactor close to the foamable sample was used as a source of an input signal for the feedback system. The latter unit was based on a PID controller TRM-210 (Oven company, Moscow, Russia). This assembly made it possible to control the temperature in the reactor with the error no worse than ±0.1 °C. The current pressure in the reactor during depressurization was measured using an APZ3421 precision pressure sensor (PIEZUS, Moscow, Russia) with the error not worse than ± 0.05 MPa. The data recordings from the pressure sensor were synchronized with video recordings from the CMOS camera (1). In addition to recording the current speckle dynamics, the video of the expanding foam was recorded through a pair of opposite side windows using an additional CMOS camera (7) with a macro lens and white light illumination (8, a halogen lamp). In this case, the scattered laser radiation was blocked using a bandpass blue-and-green filter (ThorLabs, Newton, New Jersey, USA) (9, FGB-39 type, ThorLabs, the bandpass region is 360–580 nm) located in front of the macro lens of the second CMOS camera (ToupTek Photonics company, Hangzhou, P.R. China) (7, XCAM1080PHB, the frame rate is 30 fps).

**Figure 2:**
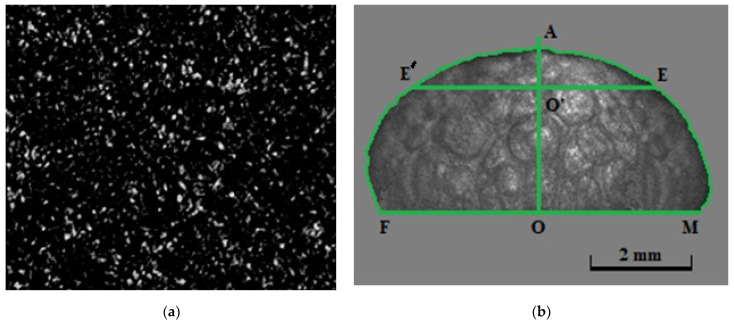
(**a**) An example of a snapshot of the dynamic speckle pattern obtained using the camera 1; the frame rate is 120 fps and the size of the image fragment is 330 × 250 pixels; (**b**) an example of the contoured image of the expanding foam.

The values of the current volume of the expanding foam were estimated in the post-processing mode using the image processing procedure previously described in [30]. Figure 2 presents the examples of captured images from channel 1 (recording of the dynamic speckle patterns, (a) and channel 2 (recording of the current images of the expanding foam, (b). In Figure 2b, the green-contoured image fragment corresponds to the visible part of the expanding foam currently located above the upper boundary of the sample container (marked by the baseline FM) and observed in the transillumination mode (Figure 1). Following [30], we can assume that the current shape of the visible part of foam (above the upper boundary of the container) can be approximated by an axisymmetric body with a cross-section defined by a contoured image fragment (Figure 2b). The current foam volume can be approximately calculated as

(1) $$V_{f}\left(t\right)\approx \pi K_{L}^{3}{\sum_{i=1}^{i_{\max}}\left[\frac{N_{i,r}\left(t\right)+N_{i,l}\left(t\right)}{2}\right]}^{2}+V_{c},$$

where $i_{\max}$ is the number of pixels along the symmetry axis (OA) from the baseline (FM) to the contour vertex, $N_{i,l}$ and $N_{i,r}$ are the numbers of pixels along the horizontal intervals (O’E’ and O’E) from the OA axis to the contour edges at the *i*-th pixel level, and $V_{c}\approx$ 80 mm^3^ is the initial volume of the plasticized polymer in the container 6. The scale factor $K_{L}$ is determined by magnification of the used macro lens and the pixel size of the camera (7). During foaming, the foam expansion factor $V_{f}\left(t\right)/V_{c}$ increased from ≈1 (start of the nucleation stage) to ≈8–10 (stabilization of the foam structure). Expressed scattering of laser radiation with pronounced speckle dynamics, caused by the transition from the nucleation stage to the intense foam expansion, took place for the used detection geometry at values of the expansion factor exceeding ≈2.

### 2.2. Principle of SSH Mapping

**Figure 3:**
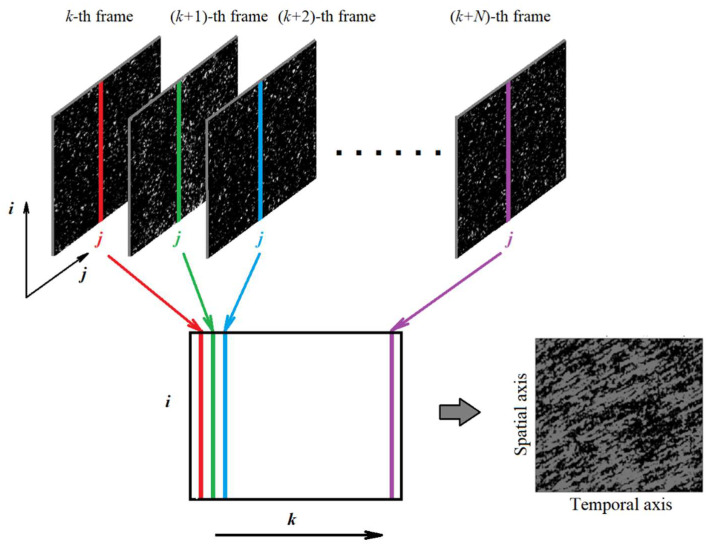
Recovery of a SSH map.

Figure 3 illustrates the principle of synthesizing a SSH map using a time-limited sequence of snapshots of time-varying speckle patterns. Stacking of the same horizontal or vertical lines from sequential snapshot frames provides a spatial–temporal map with the texture characterizing not only the qualitative features of the speckle dynamics (absence or existence of the drift component) but also such quantitative parameter as the lifetime of speckles. The average lifetime of speckles is directly related to the average length of projections of traces corresponding to individual speckles onto the horizontal (time) axis. A necessary condition for the recovery of an informative SSH map that adequately characterizes the features of speckle dynamics is a significantly shorter sampling time when recording a sequence of speckled snapshots compared with the average speckle lifetime. Another condition is that the average speckle area in the detection plane of the camera overlaps not one, but several pixels. In our case, the aperture of the camera lens was chosen in such a way that the average speckle area covered ≈ 8 pixels.

**Figure 4:**
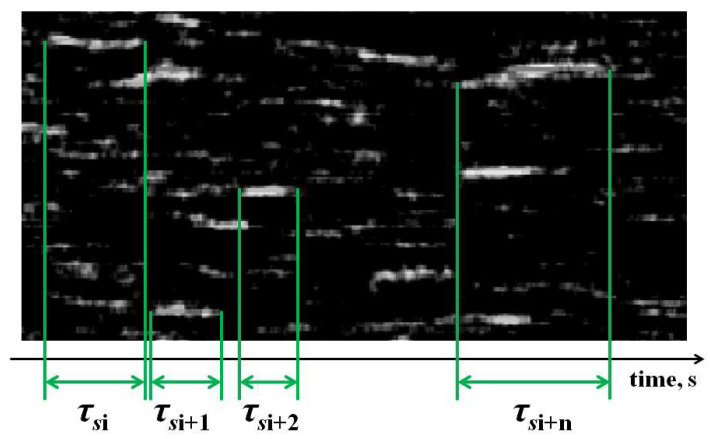
An image of a SSH map fragment illustrating a method for estimating speckle lifetimes.

A fragment of the SSH map shown in Figure 4 illustrates the principle of estimating the average speckle lifetime $\langle \tau_{s}\rangle$ for a given time interval. The $\langle \tau_{s}\rangle$ value is determined as a result of averaging over a set of $N_{tr}$ randomly chosen traces corresponding to individual dynamic speckles: $\langle \tau_{s}\rangle =\left(1/N_{tr}\right)\sum_{i}^{N_{tr}}\tau_{si}$. The start (k=s) and end (k=f) points for each trace when determining its length $\tau_{si}$ in the time domain {k} were determined from the condition $B_{i}^{k=s}=T\langle B\rangle ;B_{i}^{k=f}=T\langle B\rangle .$ Here, k is the number of a pixel belonging to the chosen i-th trace in the SSH map, $B_{i}^{k}$ is the corresponding brightness of the pixel, and 〈B〉 is the average brightness of pixels in the processed SSH map. In our case, T was taken equal to 0.1. Accordingly, the speckle lifetime for a chosen trace was estimated as $\tau_{si}=\Delta t\left(f-s\right)$, where Δt is the image sampling interval (the reciprocal of the frame rate of the camera 1). In our case, the value of $N_{tr}$ was chosen equal to 20.

The concept of estimating the average speckle lifetime for characterizing dynamic scattering systems was also considered using statistical modeling of the dynamic speckles formation. Within the framework of this approach, the current amplitude of the scattered light field at an arbitrarily chosen observation point was presented as the sum of a large number of statistically independent random phasors with the equal unit amplitudes: $E_{k}=\sum_{m=1}^{M_{ph}}\exp\left[j\left\{\varphi_{m}+\Delta \varphi_{m}\left(k\right)\right\}\right]$. Here, $\varphi_{m}$ are the initial random phases of phasors at the moment k= 0 and $\Delta \varphi_{m}\left(k\right)$ are the random phase shifts associated with the dynamic scattering of a coherent light in the examined system. Accordingly, the current intensity at the observation point can be evaluated as $I_{k}=E_{k}E_{k}^{*}={\left|E_{k}\right|}^{2}$, where the asterisk denotes complex conjugation. It should be noted that numerous works on speckle optics have repeatedly confirmed the applicability of this approach, which follows from the classical discrete scattering model, for describing the statistical properties of scattered coherent light (see, e.g., [1,2,3,31,32]).

**Figure 5:**
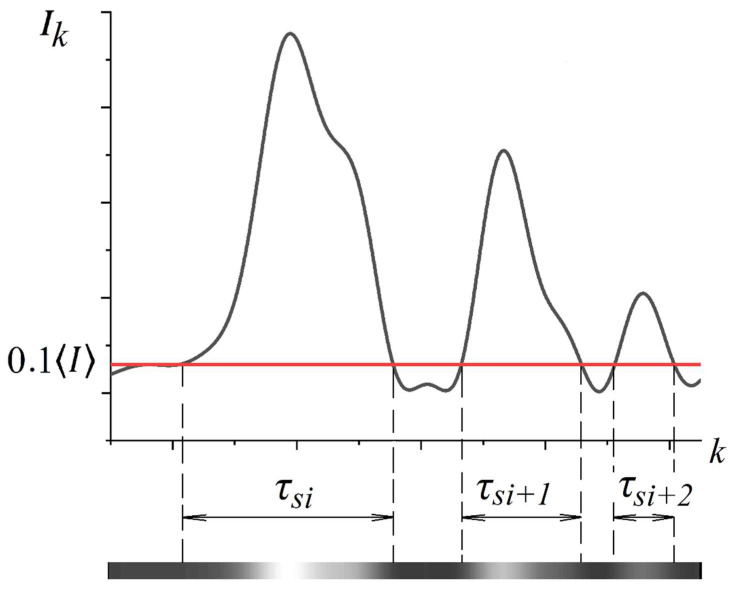
A fragment of the model dependence $\left\{I_{k}\right\}$ for an ensemble of phasors with regular phase modulation and the corresponding part of the model SSH trace for a fixed detection point.

During the simulation, random sequences $\left\{I_{k}\right\}$ of intensity values were generated for two extreme modes of accumulation of phase shifts $\Delta \varphi_{m}\left(k\right)$ with the increasing time k. In the first case, the phase shifts varied with time as $\Delta \varphi_{m}\left(k\right)=\Omega k\Delta \varphi_{m}\left(0\right)$, where $\Delta \varphi_{m}\left(0\right)$ is the initial random phase shift for m−th phasor and Ω is the rate of phase shift accumulation as the input parameter for the simulation. In the second case, the phase shifts are accumulated in the following way: $\Delta \varphi_{m}\left(k\right)=\Delta \varphi_{m}\left(k-1\right)+\Omega \phi_{m}$, where $\phi_{m}$ is a random value. In other words, case 1 corresponded to regular changes in the current phase for each phasor, while in case 2 the phases underwent random walks. The initial values $\varphi_{m}$ and $\Delta \varphi_{m}\left(0\right)$ as well as the random phase shifts $\phi_{m}$ were generated as random magnitudes uniformly distributed in the range from −π and π. The number of phasors was taken equal to 100, and the $\left\{I_{k}\right\}$ sequences with a volume of 10^7^ values were obtained for various values of the phase shift rate Ω in the range from 1.0⋅10^−3^ to 0.5. After that, the normalized autocorrelation functions of intensity fluctuations were calculated as $g_{2}\left(m\right)=\bar{\left(I_{k+m}-\bar{I_{k}}\right)\left(I_{k}-\bar{I_{k}}\right)}/{\left(\bar{I_{k}}\right)}^{2}$, where the upper line denotes averaging over the sequence. The correlation times $\tau_{c}$ of intensity fluctuations were estimated as the values of m, corresponding to the 1/e decrease in $g_{2}\left(m\right)$. In addition, the modeled values of the average lifetime of speckles were evaluated as the ratios of the total residence times of the sequences $\left\{I_{k}\right\}$ over the threshold value $0.1\bar{I_{k}}$ to half the number of threshold crossings. As an example, Figure 5 displays a fragment of the $\left\{I_{k}\right\}$ sequence in case 1 and the corresponding model SSH trace for Ω = 0.001. When displaying the model SSH trace for an arbitrarily chosen observation point, the brightest level in the gray level scale (255 bits) was selected corresponding to the value $I_{k}=3\bar{I_{k}}$.

**Figure 6:**
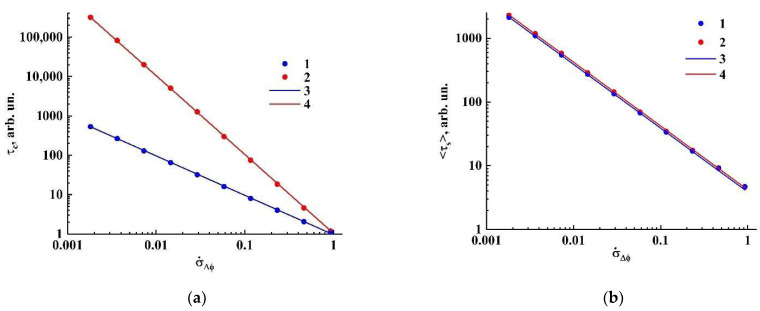
(**a**) Model values of the speckle correlation time against the root mean square of phase shifts per unit simulation step (1, 2) and the corresponding fitting dependencies (3, 4); (**b**) the same for the average speckle lifetimes. (1, 3)—an ensemble of phasors with “regular” phase dynamics; (2, 4)—an ensemble of phasors with stochastic phase dynamics.

Figure 6 displays the obtained model values of the speckle correlation time and speckle lifetime in terms of the corresponding numbers of simulation steps against the root mean square ${\dot{\sigma }}_{\Delta \varphi }$ of the random phase shifts per the unit simulation step for the considered ensembles of random phasors. Note that the ${\dot{\sigma }}_{\Delta \varphi }$ value is actually the ensemble-averaged phase shift rate. In the case of uniform distributions of phase shifts, this value is estimated as ${\dot{\sigma }}_{\Delta \varphi }=\Omega \pi /\sqrt{3}$. The model data $\langle \tau_{s}\rangle =f\left({\dot{\sigma }}_{\Delta \varphi }\right)$ in both cases admit with a high accuracy an inverse linear approximation $\langle \tau_{s}\rangle \approx A/{\dot{\sigma }}_{\Delta \varphi }$ with close values of A (Figure 6b, solid blue (3) and red (4) lines, $A_{3}=$ 3.870 ± 0.016, $A_{2}=$ 4.194 ± 0.014). At the same time, the correlation times (Figure 6a) exhibit completely different decreasing trends with the increase in ${\dot{\sigma }}_{\Delta \varphi }$ (the inverse linear decay $\tau_{c}=\left(0.966\pm 0.002\right)/{\dot{\sigma }}_{\Delta \varphi }$ in case of the ensemble of phasors with the “regular” phase modulation (1, 3), and the inverse quadratic decay $\tau_{c}=\left(1.048\pm 0.003\right)/{\dot{\sigma }}_{\Delta \varphi }^{2}$ in the case of stochastic phase shifts between the sequential simulation steps (2, 4)).

**Figure 7:**
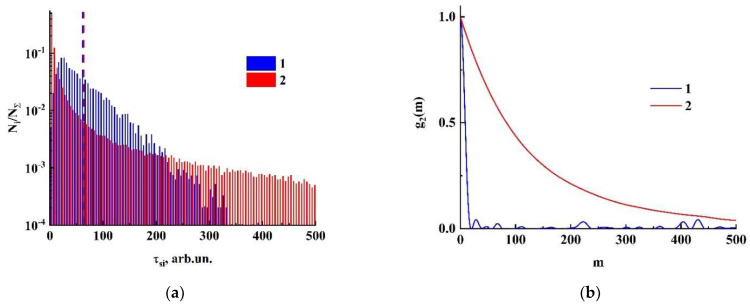
Histograms of the speckle lifetimes (**a**) and the corresponding normalized correlation functions of intensity fluctuations (**b**) for the considered ensembles of phasors. Assignment of the markers (1, 2) is the same as in Figure 6.

At first glance, this feature in the behavior of speckle correlation times and speckle lifetimes for systems with different phase dynamics seems surprising and even somewhat inconsistent. Nevertheless, the analysis of the statistics of speckle lifetimes for both model systems validates this result. As an example, Figure 7a displays the histograms of speckle lifetime distributions for both systems characterized by the same value of ${\dot{\sigma }}_{\Delta \varphi }=$ 7.0⋅10^−2^; the dashed red and blue lines mark the corresponding average lifetimes of speckles. We can conclude that, in the case of the system with stochastic phase modulation (2), the probability of the appearance of long-lived speckles is significantly larger compared with the system with regular phase dynamics (1). This causes a sufficiently slower decay of intensity correlations and, accordingly, a longer correlation time for the ensemble of phasors with the stochastic phase modulation (Figure 7b). On the other hand, in this case the high probability of the appearance of short-lived speckles (a peak near the origin in the histogram 2) causes the significantly shorter lifetime compared with the correlation time. Thus, it can be concluded that the average speckle lifetimes are invariant with respect to the type of dynamics of phase variations in stochastically interfering light waves. Accordingly, in the absence of a priori knowledge about the type of scatter dynamics, estimates of this parameter may be more preferable for characterizing microscopic motions in scattering random media as compared with the speckle correlation time. Thus, the main result of the performed modeling is that the product of the ensemble-averaged phase shift rate ${\dot{\sigma }}_{\Delta \varphi }$ by the average speckle lifetime $\langle \tau_{s}\rangle$ is a dimensionless quantity $ℵ\left(I_{th}\right)$ determined only by the threshold intensity value $I_{th}$ when estimating the speckle lifetime:

(2) $${\dot{\sigma }}_{\Delta \varphi }\cdot \langle \tau_{s}\rangle =ℵ\left(I_{th}\right).$$

### 2.3. Used Materials and Experimental Conditions

In the foaming experiments, amorphous D,L-polylactide (PURASORB PDL 04, the CAS number 26680-10-4, product # 1824008 (Corbion, Amsterdam, the Netherlands)) was used as a plasticized/foamed material. PURASORB PDL 04 is a GMP grade copolymer of D,L-lactide with the inherent viscosity midpoint of 0.4 dl/g. It is supplied in the granular form and primarily used in biomedicine and drug delivery. The reagent grade carbon dioxide (99.998% purity) was applied as the plasticizing/foaming agent. The pressure and temperature of carbon dioxide for plasticization were chosen above the critical values (≈ 8.0 MPa and ≈ 40.0 °C, respectively). Plasticization of the raw polymer was carried out for 1 h, after which the “polylactide–carbon dioxide” system was depressurized in accordance with scenarios of rapid and slow pressure release. In the first case, the pressure in the reactor was reduced from the initial value to the atmospheric value with the average rate of ≈ 0.03 MPa/s, and the frame rate of the camera (1) was chosen equal to 120 fps. Slow depressurization was performed with a significantly smaller pressure release rate (approximately 0.006 MPa/s). Accordingly, the frame rate used for capturing dynamic speckle patterns was established equal to 60 fps. Foaming experiments were carried out five times for each of the two scenarios, and the resulting data in the form of SSH maps and sequences of images of expanding foam samples were subsequently used to establish a relationship between the microscopic and macroscopic dynamics of expansion.

## 3. Results and Discussion

**Figure 8:**
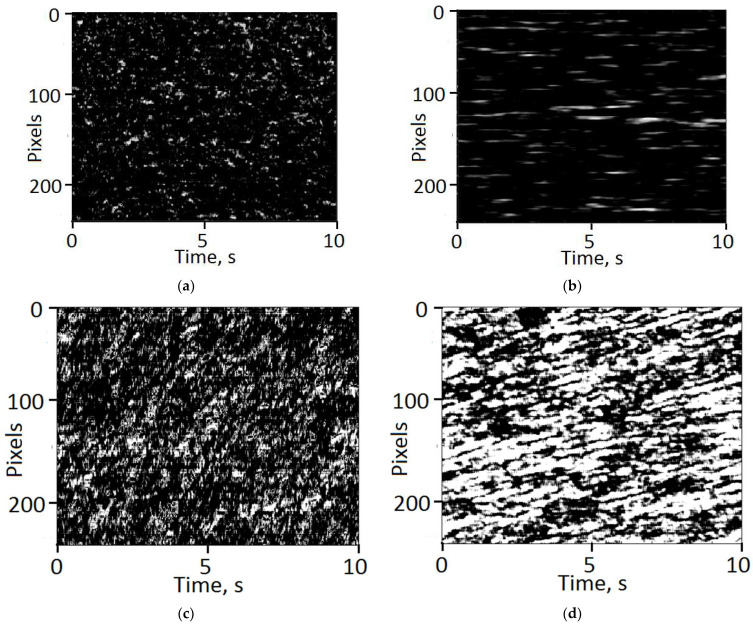
Examples of the recovered SSH maps in the cases of rapid (**a**,**b**) and slow (**c**,**d**) pressure release.

Figure 8 displays typical examples of the recovered SSH maps for the examined depressurization modes. Panels “a” and “c” correspond to the stage of intensive expansion of the foam, when the pressure in the reactor is approximately half of the initial pressure. Panels “b” and “d” display the spatial–temporal dynamics of speckles for the stage of transition from intensive expansion to stabilization of the foam structure (when the pressure in the reactor approaches the atmospheric pressure). In all cases, the time intervals for recovering the SSH maps were chosen equal to 10 s.

The following dramatic differences between the speckle structures recovered for the cases of rapid and slow depressurization can be highlighted:

− The lengths of the traces corresponding to individual speckles are several times shorter in the case of rapid depressurization as compared with the case of slow depressurization; accordingly, this indicates significantly shorter speckle lifetimes in the first case;
− The speckle traces in the case of slow depressurization are significantly inclined with respect to the time axis, which indicates a significant contribution of the regular, drift component to the speckle dynamics.

Consideration of the obtained data on the speckle lifetime at the stages of intensive foam expansion and during transition to the stabilization stage, and on the dynamics of the foam volume expansion allowed us to suggest the existence of a well-defined relationship between the microscopic and macroscopic dynamics of the expanding foam. The relationship between such parameter of the microscopic dynamics of local polymer–gas interfaces in the expanding foam as the ensemble-averaged interface mobility and the current foam volume can be considered in the framework of the following semi-quantitative model. The ensemble-averaged interface mobility can be expressed as $d\langle R_{c}\rangle /dt$, where $\langle R_{c}\rangle$ is the current ensemble-averaged size of cells (bubbles) in the expanding foam. $\langle R_{c}\rangle$ relates to the current volume of the expanding foam $V_{f}$ as $\langle R_{c}\rangle ={\left(V_{f}/KN_{c}\right)}^{1/3}$, where $N_{c}$ is the number of cells in the foam volume, and K is the scale factor determined by the cell shape (in particular, K≈ 4.19 for the near-spherical cells). Therefore, the ensemble-averaged interface mobility is expressed as

(3) $$\frac{d\langle R_{c}\rangle }{dt}=\frac{1}{3K}{\left(\frac{V_{f}}{KN_{c}}\right)}^{-\frac{2}{3}}\left\{\frac{1}{N_{c}}\left(\frac{dV_{f}}{dt}\right)-\frac{V_{f}}{N_{c}^{2}}\left(\frac{dN_{c}}{dt}\right)\right\}.$$

**Figure 9:**
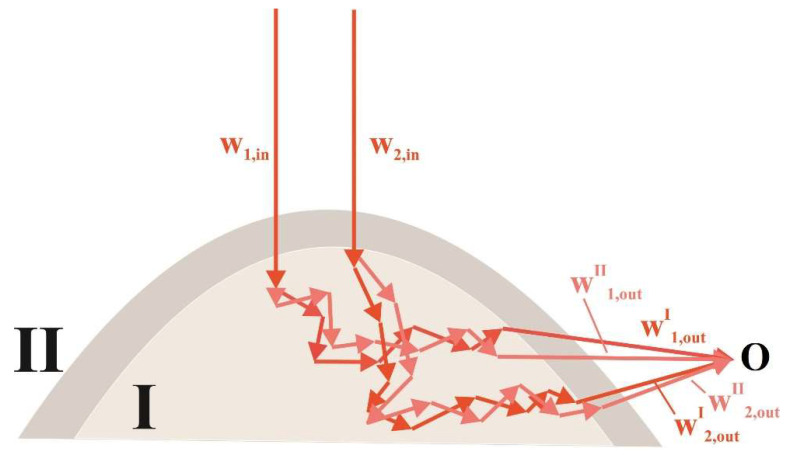
The schematic sketch illustrating the considered semi-quantitative model.

On the other hand, based on the simulation results presented above, we can consider the following scheme for the formation of a fluctuating speckle field upon multiple scattering of laser radiation in an evolving foam (Figure 9).

The current value of the intensity at the observation point O is the result of the interference of a large number of statistically independent partial waves propagating along random trajectories in the foam volume. Figure 9 schematically represents a pair of such waves ($w_{1},w_{2}$) undergoing random phase shifts due to sequences of scattering events and interfering at the point O. These waves arriving at the observation point are associated with an ensemble of random phasors similar to that considered in the numerical model in Section 2.2. During the transition of the foam from state I to state II in the course of expansion, each phasor corresponding to a partial wave acquires a phase shift $\Delta \varphi_{i}=\sum_{s}^{N_{sc,i}}\Delta \varphi_{i}^{s}$ due to microscopic structural rearrangements in the foam volume. Here, $\Delta \varphi_{i}^{s}$ is the local phase shift associated with s-th single scattering event in the sequence, and $N_{sc,i}$ is the total number of scattering events for a considered partial wave. Based on the random nature of the scattering system under consideration, we can assume the statistical independence of the local phase shifts $\Delta \varphi_{i}^{s}$ with a zero ensemble-averaged value ${\langle \bar{\Delta \varphi_{i}^{s}}\rangle }_{i}=$ 0 (the upper line denotes averaging over the sequence of scattering events for a partial wave indexed by i and the broken brackets denote averaging over the ensemble of partial waves. In this case, the application of the central limit theorem to the sums of random values $\sum_{s}^{N_{sc,i}}\Delta \varphi_{i}^{s}$ leads to the following relationship between the root mean square values $\sigma_{\Delta \varphi }$ and $\sigma_{\Delta \varphi^{s}}$:

(4) $$\sigma_{\Delta \varphi }\approx {\left(\langle N_{sc}\rangle \right)}^{0.5}\sigma_{\Delta \varphi^{s}}$$

Here, $\sigma_{\Delta \varphi^{s}}$ is the root mean square of the phase shift per unit scattering event during the transition of the system from state I to state II and $\langle N_{sc}\rangle$ is the ensemble-averaged number of scattering events for a set of partial waves incoming to the observation point. On the other hand, local phase shifts $\Delta \varphi_{i}^{s}$ per single scattering events are associated with changes in the distances $\Delta d^{s}$ between scattering centers involved in the corresponding events: $\Delta \varphi_{i}^{s}=2\pi \Delta d^{s}/\lambda$, where λ is the wavelength of the probing radiation in the probed medium. Accordingly, $\sigma_{\Delta \varphi^{s}}=2\pi \sigma_{\Delta d^{s}}/\lambda$. Following from the stochasticity of the scattering process in the probed system and the random nature of the system, we assume that the probability density function of $\Delta d^{s}$ values is zero-centered and symmetric about the origin. In this case, the ensemble-averaged absolute value $\langle \left|\Delta d^{s}\right|\rangle$ is proportional to $\sigma_{\Delta d^{s}}$ with a proportionality coefficient determined by the shape of the probability density function $\rho \left(\Delta d^{s}\right)$. In expanding foams, the average distance between the scattering centers $\langle d^{s}\rangle$ is proportional to the current average size $\langle R_{c}\rangle$ of cells: $\langle d^{s}\rangle \propto \langle R_{c}\rangle$ (see, e.g., [14]). Within the framework of the discussed semi-quantitative phenomenological model, we can consider the relationship: $\sigma_{\Delta d^{s}}\propto \left(d\langle R_{c}\rangle /dt\right)\Delta t_{I,II}$, where $\Delta t_{I,II}$ is the time interval corresponding to the transition of the system from state I to state II. Accordingly, we can write the following equation:

(5) $$\sigma_{\Delta \varphi }\propto \left(2\pi /\lambda \right)\cdot {\left(\langle N_{sc}\rangle \right)}^{0.5}\cdot \left(d\langle R_{c}\rangle /dt\right)\Delta t_{I,II}$$

Note that the product $\left(2\pi /\lambda \right)\cdot {\left(\langle N_{sc}\rangle \right)}^{0.5}\cdot \left(d\langle R_{c}\rangle /dt\right)$ can be interpreted as the ensemble-averaged rate of changes in the total phase shifts for the considered set of partial waves associated with random phasors. Setting $\Delta t_{I,II}$ equal to $\langle \tau_{s}\rangle$ and using Equation (2), we arrive at a semi-quantitative relationship between $\langle \tau_{s}\rangle$ and $d\langle R_{c}\rangle /dt$:

(6) $$\langle \tau_{s}\rangle \propto \frac{\lambda }{\frac{d\langle R_{c}\rangle }{dt}{\left(\langle N_{sc}\rangle \right)}^{0.5}}$$

As follows from Equation (6), the relationship between $\langle \tau_{s}\rangle$ and $V_{f}$ is governed not only by the dependence of $d\langle R_{c}\rangle /dt$ on $V_{f}$ and $N_{c}$, but also by the dependence of $\langle N_{sc}\rangle$ on these values. A detailed examination of the exact quantitative relationship between the ensemble-averaged number of scattering events, the foam volume, and the number of cells in the foam is a rather sophisticated problem that goes beyond the scope of this work. However, the qualitative relationship between $\langle N_{sc}\rangle$, $V_{f}$, and $N_{c}$ can be considered within the framework of general concepts of radiation transfer in random media and the optical properties of foamed substances. On the one hand, $\langle N_{sc}\rangle$ depends on the characteristic size $L_{f}$ of a foam and the transport mean free path $l^{*}$ of light propagation in the foam as $\langle N_{sc}\rangle \propto {\left(L_{f}/l^{*}\right)}^{\beta }$, where the exponent β depends on the light propagation mode in the medium. Following [33], it can be assumed that β is about 1 in the case of low-step scattering and tends asymptotically to 2 when transferring to the diffusion mode of light scattering. On the other hand, $l^{*}\propto \langle R_{c}\rangle$ and $L_{f}\propto {\left(V_{f}\right)}^{\frac{1}{3}}$. Combining these relationships, we can obtain that ${\left(\langle N_{sc}\rangle \right)}^{0.5}\propto {\left(N_{c}\right)}^{\frac{\beta }{6}}$. Thus, even in the case of a transition to a diffusion mode of light scattering in the expanding foam and significant changes in $N_{c}$ during the expansion process, the influence of this parameter is limited due to small values of the corresponding exponent approaching 1/3 in the extreme case.

A relatively simple case of the relationship between $\langle \tau_{s}\rangle$ and $V_{f}$ takes place if the number of cells $N_{c}$ is constant or changes insignificantly during the expansion:

(7) $$\langle \tau_{s}\rangle \propto V_{f}^{\frac{2}{3}}{\left(\frac{dV_{f}}{dt}\right)}^{-1}$$

Thus, in this case we can expect an approximately inverse linear dependence of the speckle lifetime on the parameter $\Psi =V_{f}^{-\frac{2}{3}}\left(dV_{f}/dt\right)$, which can be recovered from the empirical data on the time-dependent foam expansion. In the common case, the relationship between the average lifetime of speckles and the foam parameters $V_{f}$ and $N_{c}$ has the following form:

(8) $$\langle \tau_{c}\rangle \propto {\left(N_{c}\right)}^{\frac{-\beta }{6}}{\left(V_{f}\right)}^{\frac{2}{3}}{\left\{\left(\frac{dV_{f}}{dt}\right)-\frac{V_{f}}{N_{c}}\left(\frac{dN_{c}}{dt}\right)\right\}}^{-1}.$$

**Figure 10:**
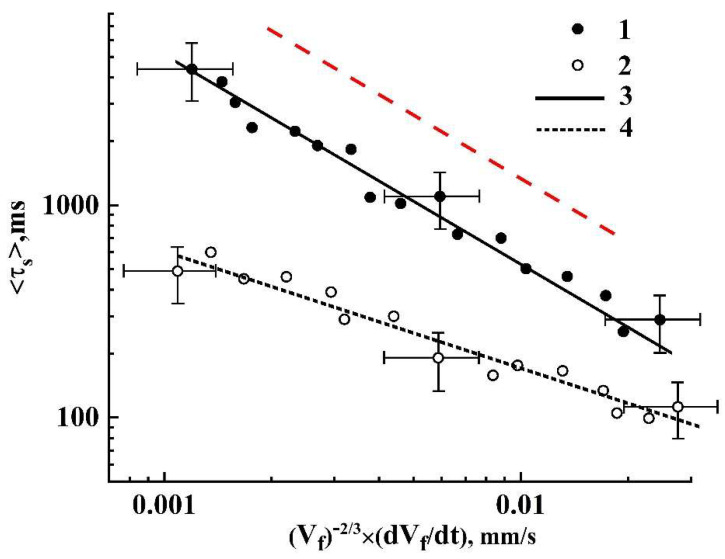
The average lifetime of speckles against Ψ in the cases of slow (1) and rapid (2) depressurization. Dependencies (3, 4) are the power-law fits $\langle \tau_{s}\rangle =C\Psi^{\alpha }$ for the datasets (1, 2). $\alpha_{3}\approx$ −(0.989 ± 0.059); $C_{3}\approx$ (5.455 ± 2.062) ms⋅(mm/s)^-^^α^_3_; $\alpha_{4}\approx$ −(0.571 ± 0.039); $C_{4}\approx$ (12.426 ± 3.058) ms⋅(mm/s)^-^^α^_4_.

Figure 10 displaying the values of $\langle \tau_{s}\rangle$ against Ψ for the examined cases of slow and rapid depressurization illustrates the trends in the behavior of the average lifetime of dynamic speckles depending on the applied depressurization mode. Selectively shown error bars correspond to the confidence level of 0.9 and indicate a statistical spread of the data obtained in the series of foaming experiments for two used depressurization modes. The red dashed line corresponds to the dependence $\langle \tau_{c}\rangle \propto \Psi^{-1}$ and serves as a guide for the eye. It is clearly seen that the trend in behavior of the dataset corresponding to slow release of the pressure is in a good agreement with the inverse linear dependence of the lifetime $\langle \tau_{c}\rangle$ on Ψ. Accordingly, we can assume that $N_{c}\approx const$ during the slow expansion of the foam. On the contrary, in the case of rapid depressurization, there is a significant deviation of the behavior of the dependence $\langle \tau_{c}\rangle =f\left(\Psi \right)$ from the inverse linear law (presumably due to the influence of the first time derivative of $N_{c}$ in Equation (8)). At the same time, this dependence allows a power-law approximation $\langle \tau_{c}\rangle \propto \Psi^{\alpha }$ with the exponent −1<α<0. In particular, this value is approximately equal to −0.57 in the case of rapid depressurization (the dataset 2 and the fitting curve 4 in Figure 10).

Following from the previously published results [30] on polylactide foam formation for two extreme depressurization modes (the “quasi-adiabatic” foaming under rapid depressurization and “quasi-isothermal” foam expansion in the case of slow pressure release), we can draw a qualitative physical picture explaining the observed features. A global difference between the quasi-adiabatic and quasi-isothermal foaming modes is that the latter mode can be considered as a sequence of gradual transitions between the close quasi-equilibrium states of the system “polylactide foam–surrounding carbon dioxide”. Formation of an ensemble of cell nuclei occurs mainly at the stage of nucleation before the foam expansion, and the number of cells in the probed slowly expanding foam varies insignificantly. In other words, the stages of nucleation and intensive foam expansion are separated in time and practically do not overlap.

On the contrary, rapid depressurization causes a substantially non-equilibrium expanding polylactide foam, for which, at each moment of time, the concentration of carbon dioxide in the polymer is excessive in relation to the current external pressure. This leads to generation of additional cell nuclei in the polymer component of the expanding foam (i.e., the nucleation and foam expansion stages overlap). Thus, such a qualitative consideration suggests that in the slow foaming mode, the time-dependent displacements of scattering centers (local interfaces) in the foam volume are characterized by a remarkable drift-like (regular) component. On the contrary, rapid expansion of the foam leads to the dominating stochastic (diffusion-like) microscopic motions of the scattering sites. Occurrence of a remarkable contribution of the drift-like component in the case of slowly expanding foams manifests itself in the textures of the recovered SSH patterns (Figure 8c,d) as the common slopes of traces corresponding to the single speckles with respect to the time (horizontal) axis. These slopes are associated with a sufficient contribution of the translational component to the speckle dynamics. In turn, this translational component corresponds to a relative macroscopic stability of the scattering structures over the observation time.

Absence of the slopes of speckle traces in the case of rapid expansion of the polylactide foam (Figure 8a,b) indicate only the boiling-type speckle dynamics associated with almost stochastic diffusion-like microscopic dynamics of the scattering units in the expanding foam volume. Such kind of dynamics is resulted in a strongly fragmented and highly irregular structure of the synthesized highly porous polylactide matrices [30].

## 4. Conclusions

It can be concluded that the speckle-based sensing of microscopic dynamics in expanding polymer foams in the version of stacked speckle history recovery exhibits a fairly high sensitivity to the features of structural rearrangements in the foam, depending on conditions of its formation. The average length of the traces corresponding to individual speckles on the recovered speckle history maps characterizes the average lifetime of dynamic speckles and, accordingly, mobility of the interphase boundaries in the expanding foam at a given stage. Occurrence of a systematic slope of individual traces with respect to the time axis on the recovered maps indicates a significant contribution of the translational component to the dynamics of the recorded speckles. In turn, this indicates close-to-regular dynamics of foam expansion at the macroscopic level, in which topological features of the foam are retained for a sufficiently long time during the expansion process. This peculiarity is associated with slow foaming, in which the stages of formation of a stochastic ensemble of cell nuclei and their subsequent growth in the formed ensemble are definitely separated in time. At the same time, an increase in the depressurization rate leads to overlapping in time of the nucleation and intensive expansion stages due to the excessive concentration of carbon dioxide in the polymer in the case of a rapidly formed non-equilibrium foam. A combined action of these processes (expansion of already existing cells and appearance of new cells in the polymer component of the foam) at the stage of a rapid increase in the foam volume leads to significant changes in the topological characteristics of the growing foam. In particular, these changes are associated with an increase in the total number of polylactide-CO_2_ interfaces in the volume of foam. As a result, only the boiling dynamics of speckles is observed upon multiple scattering of laser radiation in the rapidly expanding polymer foams.

This interpretation of the obtained speckle history maps is supported, in particular, by the dependences of the speckle lifetime on the parameter describing the generalized dynamics of interphase boundaries in the foam volume. Speckle dynamics in the case of slow depressurization is characterized by an approximately inverse linear dependence of the speckle lifetime on the generalized parameter of the foam growth, which, in the framework of the considered semi-quantitative model, corresponds to a constant number of cells in the expanding foam volume. On the contrary, the speckle lifetime in the case of rapid depressurization is characterized by a weaker dependence on the generalized parameter. In accordance with the model, this indicates an increase in the number of cells in the foam volume during its expansion. At the same time, the characteristic lifetimes of speckles in the case of fast expansion turn out to be significantly shorter in comparison with slow expansion due to significantly higher mobility of the polymer–gas interfaces in the foam volume.

In our opinion, the obtained results can be useful not only for further development of the speckle-based sensing techniques applied to control the synthesis of highly porous biomedical materials, but also for a better understanding of the fundamental features of phase separation processes in evolving complex systems.

## Data Availability

Not applicable.
